# Jottings

**Published:** 1910-02-05

**Authors:** 


### Jottings.

The Salford Guardians have decided to appoint a visiting oculist to the Hope Hospital and Cottage Homes.

New rooms for the Bradford Hospital Fund Committee were formally opened last week. The Committee has raised £6,000 a year for the city hospitals.

There are 773 persons over 70 years of age in the West Derby Union's institutions. Of this number 511 are considered as not being mentally or physically fit to take care of themselves.

The Swansea Corporation has approved of the erection of a temporary sanatorium for consumptives in the vicinity of the convalescent home. Provision is to be made for 50 beds, and the cost will approximate £4,000.

The president, Col. the Hon. Harry L. W. Lawson, M.P., M.A., will preside at the 71st annual general meeting of the Newsvendors' Institution, Tuesday evening, February 15, at Memorial Hall, Farringdon Street, London, at 7.30 p.m.

